# Multiple strategies to detoxify cottonseed as human food source

**DOI:** 10.3389/fpls.2022.1080407

**Published:** 2022-12-05

**Authors:** Yongming Liu, Yaohua Zhai, Yingge Li, Jie Zheng, Jinfa Zhang, Manoj Kumar, Fuguang Li, Maozhi Ren

**Affiliations:** ^1^ State Key Laboratory of Cotton Biology, Institute of Cotton Research of Chinese Academy of Agricultural Sciences, Anyang, China; ^2^ Institute of Urban Agriculture, Chinese Academy of Agricultural Sciences, Chengdu National Agricultural Science and Technology Center, Chengdu, China; ^3^ Hainan Yazhou Bay Seed Laboratory, Sanya, China; ^4^ Zhengzhou Research Base, State Key Laboratory of Cotton Biology, Zhengzhou University, Zhengzhou, China; ^5^ Department of Plant and Environmental Sciences, New Mexico State University, Las Cruces, NM, United States; ^6^ Chemical and Biochemical Processing Division, Indian Council of Agricultural Research (ICAR)-Central Institute for Research on Cotton Technology, Mumbai, India

**Keywords:** cotton, gossypol, detoxification, phytotoxins, natural product

## Introduction

**Figure 1:**
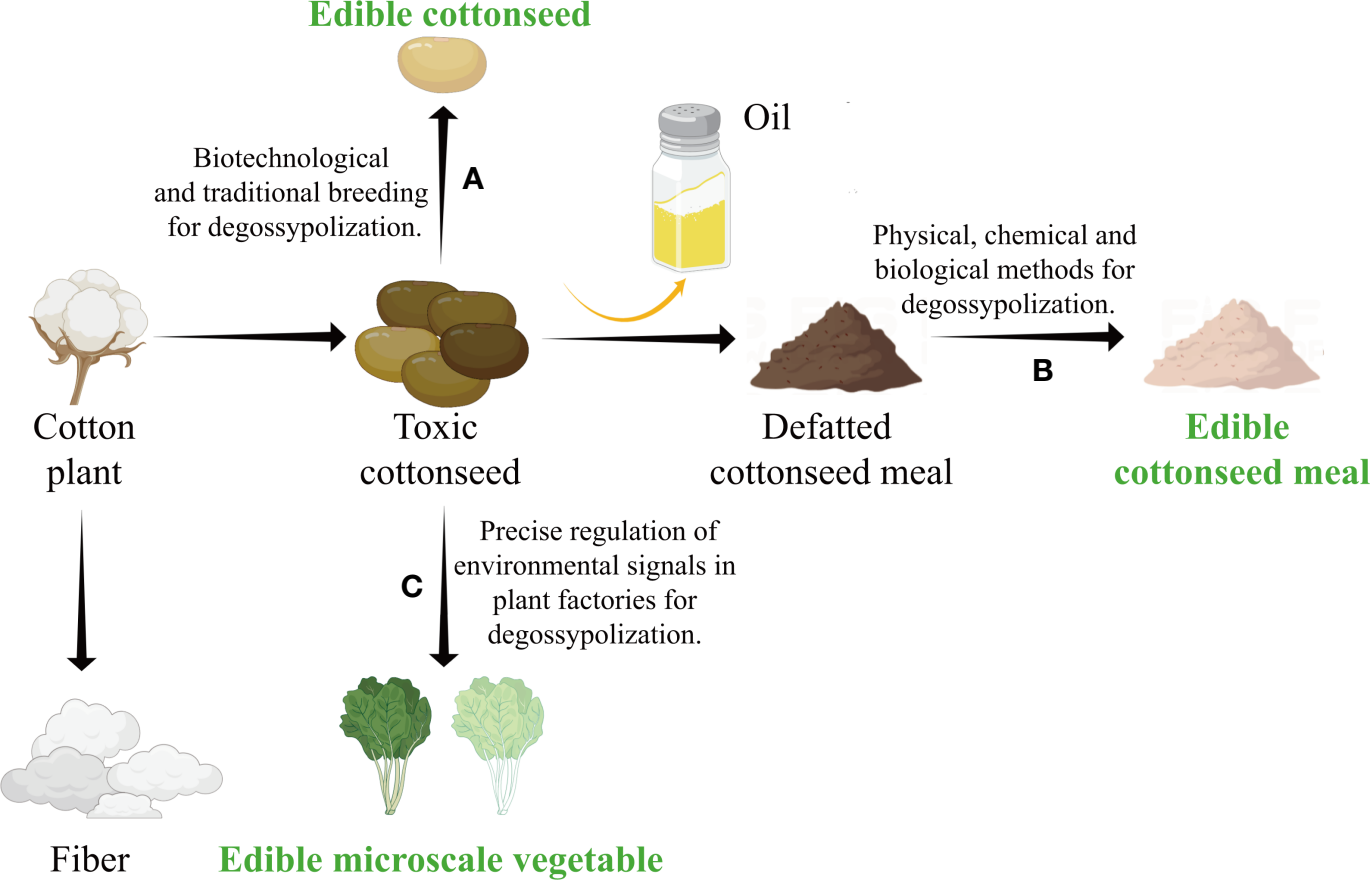
Developing edible cottonseed food with several strategies of degossypolization. **(A)** Detoxifying through conventional and molecular breeding. Creating edible low-gossypol seeds through bioengineering or conventional breeding. **(B)** Detoxifying through postharvest treatment. Producing low-gossypol cottonseed meal/protein by physical, chemical, and biological methods of degossypolization. **(C)** Detoxifying through growth conditions adjustment. Cultivating low-gossypol microscale vegetables by precise regulation of environmental factors in controlled environment. The figure is created with Figdraw.

Cotton (*Gossypium* spp.) is grown in more than 70 countries worldwide, and is the main source of natural fiber for the textile industry (Yang et al., 2020). Cotton also produces ∼1.65 kg of seed for every 1 kg of the lint fiber. Cottonseed is rich in oil (~21%) and protein (~23%), that not only makes cotton the third largest oil crop in the world, but can also meet the annual protein demand of more than half a billion people worldwide (Kumar et al., 2021). In addition, cottonseed is also abundant in other nutrients such as vitamins and minerals (He et al., 2015). Ethnic food, “Paruthi Paal” from cottonseed is regarded as “triple-nutrient” due to richness in sugars, fatty acids, and protein. Hence, relying on the development and utilization of cottonseed, cotton is expected to play an important role in alleviating the world food crisis and enriching the source of human nutrition, without extra inputs such as tillage, fertilizer, pesticide, herbicide, and irrigation. However, cottonseed is traditionally treated as a by-product, because the natural toxic gossypol greatly limits its edible value. Gossypol is a di-sesquiterpene natural-product found in cottonseed and acts as a phytoalexin in protecting plants from pests, diseases, and abiotic stresses. Nevertheless, it will seriously damage the normal functions of human respiratory system, reproductive system, and immune system when be consumed and only large ruminants such as cattle can safely digest this toxin (Gadelha et al., 2014). As a result, it not only increases the processing cost of cottonseed oil by 20%, but also hinders the use of cottonseed protein (CSP) and cottonseed meal (CSM) as human food (Zhang and Wedegaertner, 2021). The United States Food and Drug Administration (USFDA) approved the use of cottonseed containing not more than 450 ppm free gossypol for human consumption. Nowadays, cottonseed harvested in agricultural production generally exceeds the threshold and full utilizing cottonseeds to promote human nutrition and health has not yet been realized. Below, we highlighted major strategies of detoxification to promote the use of cottonseed in human food (**Figure 1**).

## Detoxifying through conventional and molecular breeding

Cotton plants synthesize gossypol mainly in roots and transport it to aboveground parts, where it is stored in the punctated brown pigment glands of stems, leaves, flowers, and bolls (Zhao et al., 2020). The ideal type for cotton production is glanded plant and glandless seed. Although such a type exists in a few wild diploid Australian cotton species including *G. bickii*, the trait has not been successfully transferred into cultivated cotton through interspecific crossing and selection. Gossypol is synthesized through the mevalonate (MVA) pathway, and a series of key enzymes involved in the synthesis process from farnesyl diphosphate (FPP) to gossypol have been characterized (Tian et al., 2018). At the same time, it is reported that the formation of cotton glands is determined by several factors including GoPGF, CGP1, CGFs, and GoSPGF (Jan et al., 2022). The loss of function of genes involved in gossypol biosynthesis or gland formation can lead to a decrease in gossypol level. Until now, several low gossypol and gossypol-free cotton glandless mutants have been reported, such as the glandless double recessive mutant (*gl_2_gl_3_*) and dominant mutant ( ${\mathrm{Gl}}_{2}^{e}$ ) (Huang et al., 2021). The underlying gene for the ${\mathrm{Gl}}_{2}^{e}$ mutant was the bHLH transcription factor GoPGF (Ma et al., 2016). These mutants are glandless in all the plant organs and are therefore vulnerable to pests and diseases. So far, low gossypol/glandless cultivars with high yield and disease resistance have been successfully developed based on mutant gene introgression (Zhang and Wedegaertner, 2021), but they are difficult to be directly applied in agricultural production. Compared with conventional breeding, seed-specific reduction of gossypol content through genetic engineering is undoubtedly a rapid way. Silencing (+)-δ-cadinene synthase *via* seed-specific RNAi reduced the level of gossypol in cottonseed by 97% without affecting the gossypol level in other parts of the cotton plant (Sunilkumar et al., 2006). At the same time, ultra-low gossypol cottonseed (ULGCS) variety showed no deleterious effects on fiber/seed yield, quality or other agronomic performance, and its stability has been confirmed by multi-year and multi-site field trials (Rathore et al., 2020). Similarly, specific silencing of the gland formation gene GoPGF in seeds decreased gossypol content by 98% and maintained normal resistance of plants to pests, with non-significant change in the levels of seed protein, oil content, fiber yield and quality (Gao et al., 2022). Moreover, the MYB transcription factor CGP1 participates in gossypol accumulation through the formation of heterodimers with GoPGF, and silencing CGP1 can also lead to a sharp reduction in gossypol levels (Gao et al., 2020). However, residual gossypol left in cottonseed after genetic engineering may also cause toxicity in human cells. Therefore, current extensive knowledge on gossypol biosynthetic pathways and evolution of genetic engineering tools such as clustered regularly interspaced short palindromic repeats (CRISPR)/Cas9 genome editing technique can be utilized efficiently to completely knock out the gossypol biosynthetic pathway in cottonseed to achieve 100% non-toxic “zero gossypol cottonseed” variety in the future.

## Detoxifying through postharvest treatment

After extraction of oil from cottonseeds, the defatted CSM containing substantial protein and toxic gossypol is left behind. Several attempts have been made using different physical, chemical, or biological methods to remove gossypol from CSM. Since gossypol is mainly stored in pigment glands, gossypol-bearing glands can be removed by physical means such as the air classification process (ACP) or liquid cyclone process (LCP) (Rathore et al., 2020). While the cottonseed flour produced by LCP was approved by USFDA as a food additive, the economic unsustainability hindered the promotion of this facility. Application of heat and pressure conditions can decrease the free gossypol concentration by more than 90% in CSM, but this method also reduces the protein content and quality (Kumar et al., 2021). In addition, gamma and electron irradiation can effectively reduce the gossypol content in CSM, and it tends to be simpler and more economical, and environmentally friendly (Kadam et al., 2021). Because gossypol can be solubilized in mixture of polar and non-polar solvents, a solvent extraction process based on acetone, ethanol, trichloroethylene, or other solutions have potential to reduce concentration of gossypol from CSM. CSP extraction achieved through alkali (potassium hydroxide) along with mixture of salts followed by ammonium sulphate precipitation resulted in ultra-low gossypol protein (Kumar et al., 2021). However, due to high processing costs, environmental pollution and food safety issues caused by residues, these chemical processes have not been successfully commercialized. Studies have shown that, based on solid-state fermentation (SSF) technology, the use of some beneficial microorganisms belonging to the genera *Aspergillus*, *Candida*, *Torulopsis*, *Mucor*, and *Rhizopus* could effectively degrade the free gossypol in CSM. Still, fermentation conditions need to be further optimized to continuously ensure high detoxification efficiency of microorganisms (Mageshwaran et al., 2018). In the future, it is necessary to develop strategies integrating physical, chemical, and biological methods, because their combinations can remove gossypol from CSM more safely and effectively (Soares Neto et al., 2021). Of course, when cotton cultivars with glanded plants and glandless seeds are commercially grown in a large scale in the future, it will render the above-mentioned processing unnecessary.

## Detoxifying through environmental modulation

Microscale vegetables, such as sprouts and microgreens, are baby plants usually produced from the seeds or other vegetative organs of vegetables, cereals, and herbs. Being small and nutritious and having a short growth cycle, microscale vegetables are suitable for indoor and vertical cultivation and play an emerging role in improving nutritional value in human diet (Kyriacou et al., 2016). Cotton seedlings have tender stems, fast growth rates with a low demand for water and fertilizer, which makes cottonseed very suitable for microscale vegetables development. However, the lack of elite germplasm for such a use, and the lack of information on nutritional values and appropriate cultivation systems are the limiting factors for the development of cotton microscale vegetables. Because glanded cottonseed contains high gossypol content, controlling the gossypol level in microscale vegetables should be reduced to a minimal level before commercialization. Recent studies have shown that light can promote the growth of cotton seedlings and effectively inhibit the accumulation of gossypol in plants (Zhang et al., 2022). Environmental controlled agricultural systems, such as plant factories and vertical farms, can reduce anti-nutrients, increase beneficial compounds, and enhance the sensory properties of plants by manipulating environmental factors (Van Delden et al., 2021), which is expected to play an important role in improving the nutrition and safety of cotton microscale vegetables.

## Conclusion

By the end of this century, the global population is expected to reach 10~12 billion, and agricultural productivity is required to increase by 50% to feed the world population (Yu and Li, 2022). Conversely, agricultural production is facing a series of challenges such as losing arable land, worsening environmental pollution and frequent extreme climatic events. Therefore, the full exploitation of the edible value of crop by-products is necessary in response to the world food crisis and human nutritional deficiencies. Cottonseed is an important by-product of cotton production, with high yield and abundant nutrition. However, the presence of gossypol in the seed makes it toxic for human consumption. Here, we propose detoxifying cottonseed through plant breeding, postharvest processing, and growth conditions controlling to meet food challenges. The improvement of cottonseed nutritional components such as oil, fatty acid and protein content has also made some progress (Wu et al., 2022). As the development of cottonseed as edible resource, it is expected to make cotton a multi-purpose (fiber, oil, food, and feed) crop. This not only opens a new way to obtain nutrition for human, but also significantly increases the net income of cotton producers.

**Figure 2:**
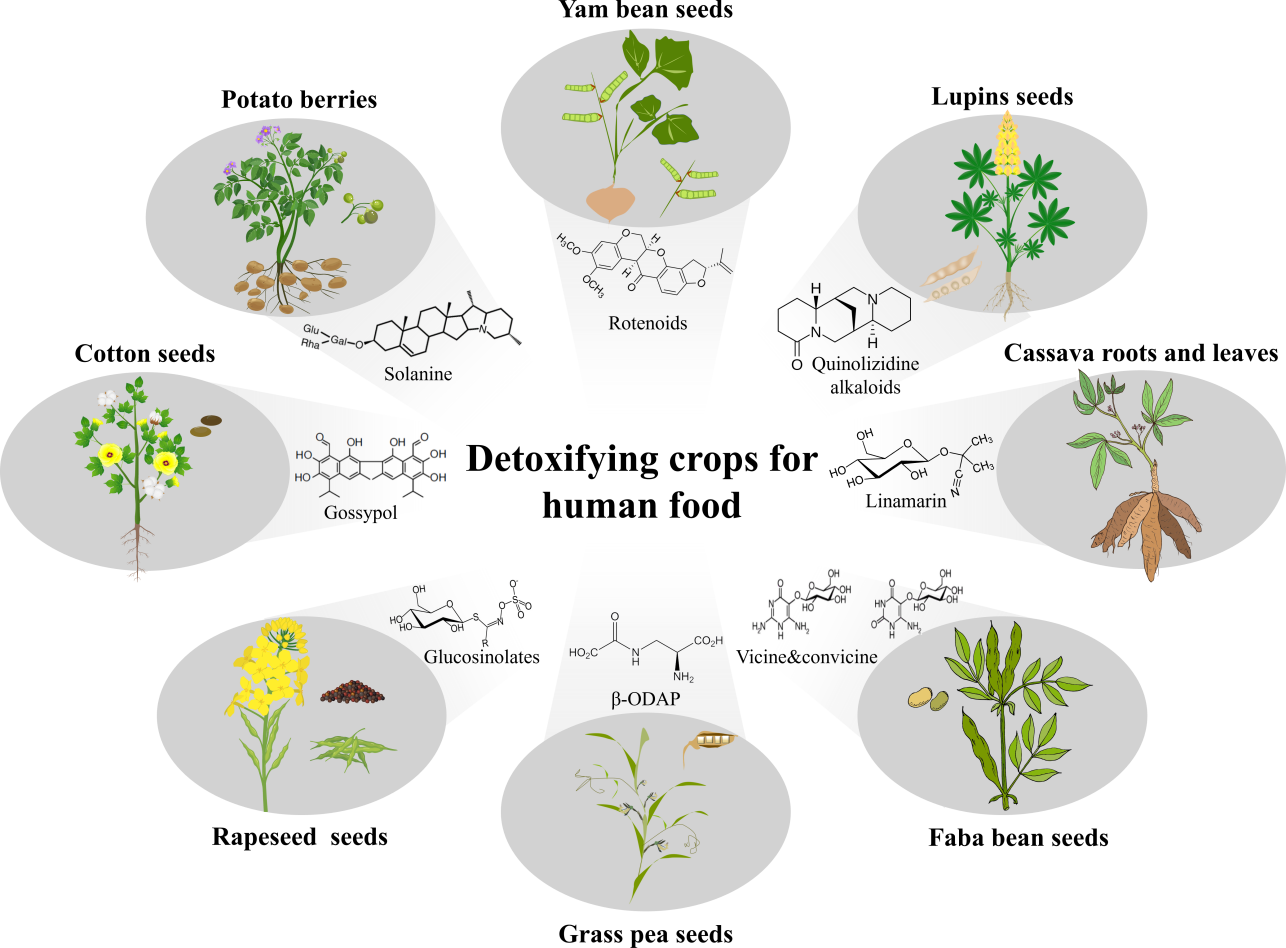
Prospects of detoxifying crops for human food. Toxic metabolites exist in many important crops, such as gossypol in cotton (*Gossypium* spp.), solanine in potato (*Solanum tuberosum*), rotenoids in yam bean (*Pachyrhizus* spp.), quinolizidine alkaloid in lupins (*Lupinus* spp.), cyanogenic glycosides in cassava (*Manihot esculenta*), pyrimidine glucosides vicine and convicine in faba bean (*Vicia faba*), β-N-oxalyl-L-α,β-diaminopropionic acid (β-ODAP) in grass pea (*Lathyrus sativus*), and glucosinolates in rapeseed (*Brassica* spp.). Expectedly, after removing phytotoxins, several new edible agricultural products, such as seeds from cotton, yam bean, lupins, faba bean, grass pea and rapeseed, berries from potato, and roots and leaves from cassava could be developed for human food. Except yam bean and grass pea, the other plant images are credited by Shanghai Hanzhong Network Technology Co., Ltd.

Like gossypol in cotton, 1,586 phytotoxins have been discovered in 844 plant species and these can be mainly divided into alkaloids, glucosinolates, cyanogenic glycosides, terpenoids, and macromolecules such as latex and proteinase inhibitors (Gunthardt et al., 2018). Proteinaceous phytotoxins, such as lectins and protease inhibitors, are not of concern in cooked food because they are thermolabile. Those antinutritional metabolites protect plant from herbivores and pathogens but is harmful to human body. Hopefully, the elimination of natural toxic metabolites in plants will help us to harvest more agricultural products as well as to enrich the kinds of crops grown (**Figure 2**). Moreover, those detoxified crops would be desirable for urban and space agriculture production because they can produce more food but less waste (Liu et al., 2021). Compared with other crop improvement strategies, detoxification represents an emerging and environmentally friendly approach for expanding food resources. It does not need to increase crop yields or change plant architecture. Therefore, to meet global food security, genetic, physical, chemical, biological or hybrid methods need to be used to remove natural toxins from more plants in the future. However, considering that these agricultural products have been considered toxic and inedible until now, more stringent safety testing by supplier and stricter supervision from government departments are needed to eliminate consumers’ concerns about the safety and promote the commercialization of these detoxified food sources.

## Author contributions

YML, YHZ, YGL, JZ, JFZ, MK, FGL, and MZR contributed to writing the manuscript. All authors contributed to the article and approved the submitted version.

## Funding

This work was supported by the National Natural Science Foundation of China (32202567), Hainan Yazhou Bay Seed Laboratory (B22C10203), Agricultural Science and Technology Innovation Program of the Chinese Academy of Agricultural Sciences (ASTIP2022QC04 and ASTIP-IUA-2022008), China Postdoctoral Science Foundation (2022M713425), and the Central Public-Interest Scientific Institution Basal Research Fund (S2022003) and Sichuan Science and Technology Program (2020JDRC0044).

## Conflict of interest

The authors declare that the research was conducted in the absence of any commercial or financial relationships that could be construed as a potential conflict of interest.

## Publisher’s note

All claims expressed in this article are solely those of the authors and do not necessarily represent those of their affiliated organizations, or those of the publisher, the editors and the reviewers. Any product that may be evaluated in this article, or claim that may be made by its manufacturer, is not guaranteed or endorsed by the publisher.
